# Exercise-Induced Blood Pressure Dynamics: Insights from the General Population and the Athletic Cohort

**DOI:** 10.3390/jcdd10120480

**Published:** 2023-11-29

**Authors:** Petra Pesova, Bogna Jiravska Godula, Otakar Jiravsky, Libor Jelinek, Marketa Sovova, Katarina Moravcova, Jaromir Ozana, Libor Gajdusek, Roman Miklik, Libor Sknouril, Radek Neuwirth, Eliska Sovova

**Affiliations:** 1 Faculty of Medicine, Palacky University, Krizkovskeho 511/8, 779 00 Olomouc, Czech Republic; petra.pesova@npo.agel.cz (P.P.); bogna.jiravska-godula@npo.agel.cz (B.J.G.);; 2 Sports Cardiology Center, Nemocnice Agel Trinec-Podlesi, Konska 453, 739 61 Trinec, Czech Republicradek.neuwirth@npo.agel.cz (R.N.); 3 Faculty of Medicine, Masaryk University, Kamenice 735/5, 625 00 Brno, Czech Republic; 4 Faculty of Medicine, University of Ostrava, Syllabova 19, 703 00 Ostrava, Czech Republic

**Keywords:** blood pressure, exercise, cardiovascular risk, prognosis, guidelines

## Abstract

Blood pressure (BP) dynamics during graded exercise testing provide important insights into cardiovascular health, particularly in athletes. These measurements, taken during intense physical exertion, complement and often enhance our understanding beyond traditional resting BP measurements. Historically, the challenge has been to distinguish ‘normal’ from ‘exaggerated’ BP responses in the athletic environment. While basic guidelines have served their purpose, they may not fully account for the complex nature of BP responses in today’s athletes, as illuminated by contemporary research. This review critically evaluates existing guidelines in the context of athletic performance and cardiovascular health. Through a rigorous analysis of the current literature, we highlight the multifaceted nature of exercise-induced BP fluctuations in athletes, emphasising the myriad determinants that influence these responses, from specific training regimens to inherent physiological nuances. Our aim is to advocate a tailored, athlete-centred approach to BP assessment during exercise. Such a paradigm shift is intended to set the stage for evidence-based guidelines to improve athletic training, performance and overall cardiovascular well-being.

## 1. Introduction

Exercise blood pressure (BP) monitoring has become a new era in cardiovascular diagnostics, particularly for athletes. It offers a broader perspective than traditional resting BP measurements, capturing the unique physiological adaptations that athletes undergo during intense physical exertion [1]. However, navigating the complex landscape of BP dynamics during exercise and distinguishing between normative and aberrant responses remains a nuanced challenge.

Historical guidelines, led by influential organisations such as the American Heart Association, have been fundamental in distinguishing typical BP responses from potential hypertensive indicators, drawing their insights predominantly from research conducted in the 1970s [2]. While these benchmarks are invaluable and based on outdated studies that were often not specifically designed for athletes, they may not fully capture the diverse cardiovascular responses of today’s elite athletes.

Advances in research and a growing understanding of the multifaceted nature of exercise BP, particularly in athletes, require a re-examination of these traditional guidelines. Misinterpretations can lead to sub-optimal training recommendations, compromising athletic performance and potentially health. The urgency to update our understanding has never been greater.

This review takes a deep dive into the complex interplay of factors that influence blood pressure during exercise, with a particular focus on athletes. By analysing the vast body of modern literature, we aim to highlight the limitations of outdated guidelines and advocate a more refined, athlete-centred approach. Our mission is to advocate a more holistic and evidence-based framework for the assessment of exercise BP to optimise both athletic performance and cardiovascular health.

## 2. Blood Pressure Responses to Exercise

In this section, we explore the multifaceted nature of the blood pressure response to exercise. Starting with a historical overview of exercise blood pressure guidelines, we consider the physiological dynamics that characterise blood pressure behaviour during exercise. We address the variability in guidelines for defining exaggerated blood pressure responses and discuss the determinants that contribute to blood pressure variability during exercise. The significance of submaximal versus peak blood pressure readings is assessed, along with their prognostic implications. Special consideration is given to the unique blood pressure dynamics observed in athletes, highlighting the need for tailored assessments. Finally, we look ahead to the evolving landscape of exercise BP dynamics, emphasising the need for future research and guideline development in this area. Each subsection is designed to provide a comprehensive understanding of the current knowledge and emerging evidence in the field and to provide a foundation for future research and practical application in sports medicine and exercise physiology.

### 2.1. Historical Perspective on Exercise Blood Pressure Guidelines

Prominent health organisations, in particular the American Heart Association (AHA), have established benchmark reference values suggesting that systolic blood pressure (SBP) should typically increase by ~10 mmHg per metabolic equivalent (MET) during graded exercise testing (GXT) [1]. These guidelines are based on landmark studies in the 1970s by Fox et al. [3] and Naughton et al. [4] in predominantly healthy young adult men.

Naughton et al. in 1973 studied 25 healthy men aged 18–29 years during treadmill testing and proposed a single reference point of SBP rising ~10 mmHg/MET at intensities of 85% of maximal effort. The AHA exercise BP guidelines are directly derived from this foundational work on exercise testing in the 1960s and 1970s.

Pioneering research by Ekblom et al. in 1968 [5] first reported the typical SBP increase of ~10 mmHg/MET during bicycle ergometry testing in healthy young men. Although powerful at the time, these studies were limited to a narrow demographic group.

Recognising this limitation, Pollock et al. in 1982 [6] specifically investigated exercise BP responses in healthy women and found differences compared with men. As early as 1992, Ogawa et al. [7] noted that most previous research on exercise BP had focused narrowly on young, trained men. They helped broaden the focus by comparing women, middle-aged and older adults.

While historically invaluable, reliance on these benchmarks from limited populations may misrepresent BP trends in contemporary diverse cohorts. For example, a 2021 study by Hedman et al. reported differential SBP responses in different age groups during exercise testing, challenging the uniform 10 mmHg/MET guideline [8].

With changing demographics, lifestyle changes and advances in medical care since the 1970s, contemporary research highlights potential limitations to the blanket application of these early guidelines. Population-based studies using improved BP monitoring techniques have shown that BP responses to exercise vary with age, fitness level and health status [2,7,9,10].

### 2.2. Physiological Dynamics of Blood Pressure during Exercise

Exercise induces a number of physiological changes, of which changes in BP dynamics are prominent. As exercise intensity increases, SBP typically exhibits a linear increase [11], reflecting the increased metabolic demands of the body and increased cardiac output. This rise in SBP, as detailed by Rowell, L.B. or Nystoriak, M.A. [12,13], is a complex interplay of factors that include not only increased cardiac output but also sympathetic activation, changes in vascular resistance and thermoregulatory responses that collectively increase blood flow to the active musculature [14].

Conversely, diastolic blood pressure (DBP) is relatively stable during exercise. Some people may even experience a slight decrease. This stability or decrease in DBP, as highlighted by Carpio-Rivera, E. et al. [15] and also by Kelley, G.A. et al. [16], is primarily due to metabolic vasodilation in the exercising muscles, ensuring adequate oxygen delivery and waste removal.

The autonomic nervous system, particularly the sympathetic branch, plays a central role in the modulation of blood pressure during exercise. For example, bursts of sympathetic activity can induce vasoconstriction in specific vascular territories, thereby influencing overall BP and directing blood flow according to the immediate needs of the body, a phenomenon discussed by Charkoudian, N. et al. and Joyner, M.J. [17,18]. At the same time, complex systems such as the renin-angiotensin-aldosterone system and endothelial function subtly but profoundly shape BP responses to exercise [19,20].

The implications of these physiological nuances go beyond academic interest. Clinicians armed with this knowledge can better predict and manage potential hypertensive episodes during rehabilitative or therapeutic exercise sessions. For patients with particular cardiovascular profiles, an in-depth understanding of BP dynamics during exercise, as recommended by Pescatello, L.S. et al. [21], can guide the design of individualised exercise programmes that maximise health benefits while minimising potential risks.

### 2.3. Different Guidelines for Defining an Exaggerated BP Response

**Figure 1:**
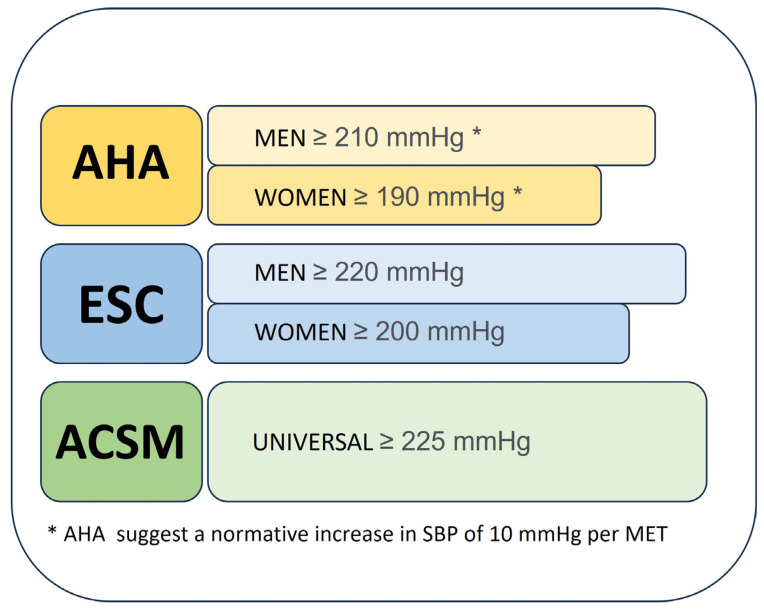
Comparative Systolic Blood Pressure Thresholds During Exercise as Recommended by Leading Health Associations. This figure graphically illustrates the recommended systolic blood pressure (SBP) thresholds during exercise by the American Heart Association (AHA), the European Society of Cardiology (ESC), and the American College of Sports Medicine (ACSM). The lengths of the bars correspond to the numerical values of the thresholds, with AHA recommending an SBP of ≥210 mmHg for men and ≥190 mmHg for women, which includes a normative increase of 10 mmHg per MET. ESC proposes slightly higher thresholds of ≥220 mmHg for men and ≥200 mmHg for women. ACSM advises a universal threshold of ≥225 mmHg, applicable to all genders. These visual bars serve to provide a clear, at-a-glance comparison of the SBP thresholds across different guidelines.

A notable aspect of cardiovascular guidelines is the variability in the definition of an abnormal blood pressure response during exercise. Different organisations suggest different thresholds (see also Figure 1):

- American Heart Association: The AHA defines a systolic blood pressure of 210 mmHg for men and 190 mmHg for women as potentially worrying during exercise, emphasising an approximate increase in SBP of 10 mmHg per MET [22].
- European Society of Cardiology (ESC): The ESC has slightly higher thresholds, recommending 220 mmHg for men and 200 mmHg for women [23].
- American College of Sports Medicine (ACSM): Taking a different approach, the ACSM proposes a unisex threshold, setting a cut-off of 225 mmHg for both sexes [24].

These guidelines are primarily aimed at identifying abnormal or worrisome blood pressure responses to stress, particularly during exercise testing. However, it’s important to remember that in certain populations, such as athletes, BP readings may naturally exceed these thresholds due to their unique physiological adaptations.

Athletes, due to their enhanced cardiovascular fitness, may have BP readings that exceed these norms without this being indicative of pathology. The challenge is to distinguish between an adaptive, athletic response and a potential health problem. With this in mind, recent research and clinical practice are moving towards more individualised assessment. Rather than rigid thresholds, some studies now use population-specific percentiles to assess whether an individual’s systolic BP exceeds, for example, the 90th percentile for their age, sex and other demographics. This tailored approach recognises the inherent variability between different groups and individuals [25].

However, the lack of a standardised guideline results in discrepancies in the reported prevalence rates of abnormal BP response during exercise, ranging from as low as 1% to as high as 61% in different studies [26,27,28]. To make matters worse, many definitions don’t take into account important confounding factors such as age, gender or fitness level. As highlighted by Benbassat, J. and Froom, P. [29], such omissions can skew interpretations and potentially misclassify individuals.

### 2.4. Determinants of BP Variability during Exercise

**Figure 2:**
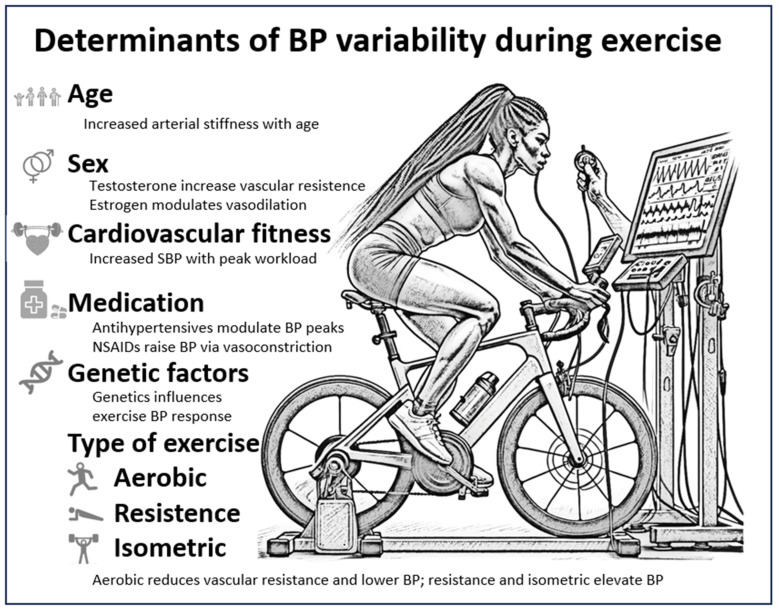
Determinants of BP variability during exercise. This schematic illustrates the factors that influence the response of blood pressure (BP) to exercise. Age affects vascular compliance by increasing arterial stiffness and affecting BP. Hormones such as testosterone and estrogen influence vascular resistance and vasodilation, respectively, and alter BP. Cardiovascular fitness correlates with higher peak systolic BP. Medication, especially antihypertensives, can modulate peak BP, while NSAIDs can increase BP by vasoconstriction. Genetic factors play a role in individual BP responses to exercise. Different types of exercise have different effects: aerobic exercise tends to reduce vascular resistance and lower BP, while resistance and isometric exercises can increase BP by increasing muscle tension. The cyclist image was generated using DALL·E 3.

Several factors may influence BP responses during exercise (Figure 2):

- Age and gender: Both age and sex have been found to be important determinants of BP dynamics during exercise. Tuka et al. [30] found that older individuals have an increased SBP response compared to their younger counterparts. This age-related increase in BP is a multifaceted phenomenon influenced by several factors. It is partly a consequence of reduced arterial wall elasticity, which may lead to increased resistance in the peripheral vasculature [31]. In addition, Trinity et al. [9] have shown that age-related changes in vascular function, such as increased arterial stiffness, contribute to the enhanced blood pressure responses observed during exercise. BP patterns in women, which are often different from those in men, may be due to hormonal variations and different vascular characteristics. For example, Smith et al. [32] noted that oestrogen is known to exert vasodilatory effects that may modulate BP responses in premenopausal women. Experimental research has further elucidated the complex interplay of various cardiovascular parameters during exercise in both sexes. These parameters include heart rate, stroke volume and vascular resistance [5,6]. In addition, Smith et al. [32] and Trinity et al. [9] have shown that the exercise pressure reflex, the body’s own mechanism for regulating blood pressure during physical activity, works differently in men and women.
- Cardiovascular fitness: An individual’s cardiovascular fitness is directly related to their BP dynamics during exercise. Higher levels of fitness are often associated with greater increases in SBP during GXT [25,33].
- Medication and medical conditions: Medication, particularly antihypertensive drugs such as beta-blockers, can significantly modulate BP dynamics during exercise, as observed by Chick, T.W. et al. [16]. It is important to note that according to Chant, B. et al. [34], despite control of baseline blood pressure, antihypertensive medication does not protect against exaggerated increases in blood pressure during peak exercise testing.
- Genetic factors: Genetics, an often-overlooked determinant, may influence exercise-induced BP responses. Certain genetic markers or hereditary predispositions may increase or decrease blood pressure variability during exercise. Studies by Rankinen, T. et al. [35], Rankinen, T. et al. [36] and Montasser, M.E. et al. [37] have all addressed this area. Although not the focus of this review, recent research suggests a genetic basis for BP variability during exercise.
- Type of exercise: The type of exercise undoubtedly plays a crucial role in determining cardiovascular responses, particularly in terms of blood pressure dynamics.

 1. Aerobic exercise (e.g., running, cycling):

Aerobic exercise, such as that performed on a treadmill or stationary bicycle, elicits distinct physiological responses. During sustained aerobic activity, there’s a marked initial increase in SBP. As exercise progresses, this increase in SBP tends to plateau or may even decrease slightly due to vasodilation in the active muscles. In contrast, DBP typically remains stable or may decrease slightly, as observed by Pescatello, L.S. et al. [21].

 2. Resistance training (e.g., weightlifting):

Resistance training, especially with heavy weights and fewer repetitions, may cause a marked transient increase in both SBP and DBP. This increase may be due to the Valsalva manoeuvre often performed during such activities, combined with increased peripheral resistance due to muscle contractions, as described by MacDougall, J.D. et al. [38].

 3. Isometric exercises (e.g., handgrip, plank):

Isometric exercises, characterised by constant muscle length and minimal joint movement, can cause significant increases in blood pressure. Activities such as sustained handgrip exercises can cause significant increases in both SBP and DBP due to the continuous muscular tension and lack of relaxation phases. Millar, P.J. et al. [39] have highlighted this phenomenon in their research.

It is important for athletes and trainers to be aware that the choice of exercise modality can have a profound effect on cardiovascular responses. For example, stationary cycling predominantly involves leg muscles with shortening contractions, leading to increased SBP. On the other hand, treadmill activities involve a wider range of muscles, including the trunk, leading to lengthening contractions and, consequently, a relatively lower peak systolic blood pressure. In addition, specific actions such as gripping the handlebars tightly during stationary cycling (an isometric contraction) may further increase blood pressure due to increased vascular resistance, as noted by Canivel, R.G. et al. [40].

Building on these established modalities, a recent large-scale meta-analysis by Edwards JJ et al. [41] suggests that isometric exercises are particularly effective in improving resting blood pressure. Their findings suggest that exercises such as isometric handgrips and wall squats rank highly for reducing SBP and DBP, respectively, and thus inform future guidelines for exercise-based antihypertensive interventions.

In practice, this means that integrating isometric exercises alongside aerobic and resistance training could provide a comprehensive approach to managing and improving blood pressure in both the general population and athletes. The nuanced response of blood pressure to different types of exercise underlines the importance of tailored exercise prescriptions for cardiovascular health.

### 2.5. The Importance of Submaximal versus Peak Blood Pressure

Historically, the field of exercise physiology and cardiology has placed considerable emphasis on the peak blood pressure values recorded during GXT. More recently, however, the focus has shifted. Studies have now highlighted the importance of exaggerated exercise BP (EEBP) as a critical indicator of cardiovascular disease (CVD) risk [42,43,44]. In this context, submaximal BP measurements taken during moderate-intensity exercise have gained prominence. Such measurements provide a more nuanced understanding of BP dynamics, capturing potential risks that may be missed when relying solely on traditional resting BP measurements [45]. In addition, they can reveal hidden hypertension, highlighting the critical role of EEBP in assessing cardiovascular health [46].

Sarma, S. et al. [47] have been instrumental in advancing our understanding of EEBP during submaximal exercise. Their findings elucidate the relationship between EEBP and increased cardiac and vascular stiffness, particularly in middle-aged women. This suggests that EEBP may serve as an early warning for conditions such as heart failure with reduced ejection fraction, which has important clinical implications. Notably, other research has shown that even modest increases in exercise systolic BP above a threshold of 150 mmHg during moderate activity are associated with hypertension. This association has been further confirmed by ambulatory BP monitoring, as highlighted by Borlaug, B.A. et al. [48]. 

Further evidence comes from Mariampillai, J.E. et al. [49], who examined the continuum of exercise BP and cardiovascular risk. Their work described a linear relationship between submaximal exercise systolic BP, specifically at a workload of 100 watts, and susceptibility to coronary heart disease. They found that beyond a systolic BP of 165 mmHg, there was a marked increase in the risk of coronary heart disease, highlighting the clinical importance of monitoring and managing EEBP in cardiovascular assessment. However, despite the growing body of evidence, much of the scientific community and guidelines remain fixated on peak BP measurements, highlighting the need for a paradigm shift [50].

### 2.6. Prognostic Significance of BP Responses

Although the precise definition of ‘exaggerated’ BP responses remains controversial, there is a consistent association between such patterns and an increased risk of latent hypertension [51]. Research has documented a 1.4- to 4.2-fold increased relative risk of hypertension onset based on these exercise-induced BP patterns [52]. Beyond just hypertension, these exaggerated responses have been correlated with adverse outcomes such as sudden cardiac death [53], cardiovascular events [52], and changes in myocardial structure [54].

Recent findings have focused on specific BP thresholds during exercise that may signal increased risk. For example, exceeding systolic BP levels of 150 mmHg during moderate-intensity exercise has been associated with latent hypertension—an association that has been further strengthened by ambulatory BP monitoring [11]. A significant study showed that a systolic BP threshold of ≥170 mmHg during the Bruce protocol stage 2 treadmill test heralded an increased risk of major adverse cardiovascular events. This association was manifested by an adjusted hazard ratio of 1.33 (1.01–1.76), highlighting the clinical implications of exercise-induced BP dynamics [55].

Schultz et al. [56] provided a landmark meta-analysis elucidating the correlation between exaggerated systolic BP during various submaximal exercises and cardiovascular disease risk in individuals with normative resting BP. Their exhaustive analysis revealed that elevated systolic BP at various submaximal exercise intensities corresponded to a significant 36% increase in cardiovascular events and mortality. In addition, each 10-mmHg increase in systolic BP during these exercises was associated with a 4% increase in event rates. Crucially, these correlations persisted even when adjusted for resting BP, age, sex, and other established CVD risk markers, highlighting the indispensable role of EEBP in shaping cardiovascular outcomes.

### 2.7. Blood Pressure Dynamics in Athletes: A Special Consideration

Due to their unique physiological adaptations and rigorous training regimens, athletes often exhibit different BP responses compared to the general population. The elevated SBP seen in athletes during exercise has been the focus of numerous studies, but its clinical implications remain a subject of ongoing debate.

Caselli et al. [57], in their study of Italian elite athletes, highlighted that exaggerated BP responses during exercise could herald a 3.6-fold increase in the risk of incident hypertension over 6.5 years. Conversely, in a distinct cohort of Olympic athletes, elevated BP responses did not correlate with adverse health outcomes, suggesting the benign nature of these responses in highly trained individuals [58].

The landscape of BP dynamics in athletes is complex. While there is little consensus on the standard SBP response to exercise in elite athletes, it remains difficult to establish a direct relationship between elevated SBP during GXT and any pathological overtones. Furthermore, the adoption of guidelines tailored to the general population may not be appropriate for athletes due to their different physiological milieu [59,60].

In the field of sports medicine, several key considerations emerge:

- Athletes should be vigilant against unwarranted gains in adiposity, given its established association with hypertension [61].
- Athletes with a family history of hypertension may benefit from more regular cardiovascular screening [62,63].
- The rate of BP escalation during GXT may provide more nuanced insights than the final value alone [64,65].
- The modality of GXT, whether bicycle ergometry or treadmill running, may influence SBP measurements. It is well documented that bicycle ergometry often records a higher SBP than treadmill testing [59,66,67].
- Furthermore, the increased SBP observed in endurance athletes is often associated with superior peak oxygen consumption (VO2max), increased work capacity and, occasionally, increased myocardial mass. The lack of evidence of adverse outcomes suggests that the elevated SBP in such athletes may be a manifestation of physiological adaptations to rigorous training rather than a pathological deviation [68,69].
- Bjarnason-Wehrens et al. [70] highlighted the nuances of exercise-induced arterial hypertension (EAH) in athletes. Their work highlighted the importance of considering various factors such as exercise intensity, duration and modality when assessing BP responses in athletes. They suggested that EAH may not always be a maladaptive response but rather an adaptive mechanism to meet the increased metabolic demands of strenuous physical activity.
- Furthermore, Hedman et al. [71] emphasised the role of the workload-indexed BP response in predicting mortality, highlighting the multifaceted nature of exercise-induced BP dynamics in athletes.
- Recent research in the field of cardiorespiratory fitness and its correlation with blood pressure dynamics introduces new parameters that may better differentiate between adaptive and maladaptive cardiovascular responses in athletes. For example, the study by Ligetvari et al. [72] sheds light on the complex interplay between apelin levels—a cardiokine/myokine—and peak exercise performance. The observed correlation between changes in plasma apelin-13 levels and key performance indicators such as maximal metabolic equivalent, relative maximal O_2_ consumption and peak aerobic performance suggests that, beyond traditional SBP measurements, a more comprehensive assessment including SBP/VO2 and SBP/W slopes, as well as aerobic performance and stroke work, may provide a more refined understanding of an athlete’s cardiovascular efficiency and adaptability. Such metrics could be invaluable in distinguishing the elevated SBP response during exercise as an adaptive mechanism associated with improved physical performance rather than a premature sign of cardiovascular stress or pathology. This nuanced perspective highlights the potential of integrating multifactorial assessments to cultivate a more accurate and athlete-centred model of cardiovascular health in sports medicine.

In conclusion, although the exaggerated BP responses in athletes during exercise are evident, their clinical implications remain to be elucidated. A holistic approach, taking into account the athlete’s training regimen, physiological adaptations and individual predispositions, is essential for an accurate assessment of cardiovascular risk in this unique population.

### 2.8. Looking Ahead: Navigating the Future Landscape of Exercise Blood Pressure Dynamics

While long-standing guidelines have provided a baseline understanding, the dynamic evolution of scientific knowledge requires a timely recalibration of our perspectives on BP responses during exercise. As we move forward, criteria need to be more attuned to the multiple factors influencing BP dynamics, potentially moving from an over-reliance on peak values to a more holistic assessment of submaximal values and the continuum of the BP gradient.

The current landscape reveals significant gaps in our understanding of the intricate mechanisms underlying exercise-induced BP changes. This underscores the need for more longitudinal, prospective studies aimed at unravelling the enigmatic intricacies of BP modulation during physical activity. A harmonised approach, emphasising the standardisation of investigative techniques and exercise protocols, may greatly enhance the robustness and reproducibility of future research efforts in this area.

The refinement of our understanding of the response of blood pressure (BP) to exercise now extends beyond traditional guidelines to include evidence from recent research on cardiac biomarkers. The work of Aengevaeren et al. [73] provides compelling evidence linking post-exercise elevations in cardiac troponin I with an increased risk of mortality and major adverse cardiovascular, signalling a paradigm shift in cardiovascular risk assessment for athletes, particularly those over 40 years of age involved in endurance sports. These findings are echoed by Omland and Aakre [74], who discuss the detectability of cTn I and T in healthy individuals and their association with chronic myocardial injury.

Juhani Airaksinen [75] emphasises the importance of understanding the transient nature of exercise-induced cTn release and its potential as an early marker of cardiovascular events and urges caution in interpretation. The review by Aengevaeren et al. [76] extends this discussion by examining the possible physiological and pathological origins of post-exercise cTn elevations and their association with occult coronary artery disease. Finally, Janssen’s study [77] broadens the perspective by examining cTn release in child and adolescent athletes, highlighting the need for future research to differentiate between benign and potentially harmful elevations.

In conclusion, emerging evidence suggests that monitoring cTn in conjunction with BP and exercise may provide a more comprehensive assessment of an athlete’s cardiovascular function and risk [78,79]. This integrative approach is critical as we develop evidence-based guidelines that incorporate the multifaceted dynamics of BP and biomarkers, ultimately improving athlete care and cardiovascular prognosis.

## 3. Conclusions

The study and interpretation of blood pressure dynamics during exercise have traditionally been approached using broad benchmarks. However, recent evidence highlights a paradigm shift towards a more nuanced understanding of the dynamic nature of BP responses, particularly during exercise. This shift is particularly pronounced in the athletic community, where the interplay of individual factors significantly influences these dynamic responses. Recognising and giving due weight to these individual determinants is not just academic; it is a practical imperative. As our understanding deepens, there is a growing consensus that close monitoring of these dynamic blood pressure responses could become a key tool in predicting early cardiovascular events or even sudden cardiac death, especially in athletes. This transformative shift is moving us towards a more refined, individualised approach to cardiovascular risk assessment and promises to revolutionise patient care and preventive strategies in the field of exercise health.

## Data Availability

The review was based on publicly available academic literature databases.
